# Long-term trends in specialized outpatient health care utilization: an analysis in the context of a primary health care reform

**DOI:** 10.1186/s12913-025-12924-1

**Published:** 2025-07-01

**Authors:** Hannes Kohnke, Andrzej Zielinski, Anders Beckman, Henrik Ohlsson

**Affiliations:** 1 Department of Clinical Sciences, Faculty of Medicine, Lund University, Lund, 370 10 Bräkne-Hoby Vårdcentral, Parkvägen 4, Bräkne-Hoby Sweden; 2 Blekinge Centre of Competence, Region Blekinge, Vårdskolevägen 5, Karlskrona, 371 41 Sweden; 3 Department of Clinical Sciences, Faculty of Medicine, Box 50332, Malmö, SE-202 13 Sweden; 4 Center for Primary Health Care Research, Department of Clinical Sciences, Lund University, Lund, Sweden; 5 Center for Primary Health Care Research, Department of Clinical Sciences, Lund University Clinical Research Centre (CRC), Box 50332, Malmö, SE-202 13 Sweden

**Keywords:** Delivery of health care, Health care utilization, Health equity, Health policy, Patient choice, Privatization, Sweden

## Abstract

**Background:**

As primary health care forms the basis of the health care system, it is regarded as an efficient way to address main causes of, and risk factors for, poor health. In the Swedish health care system, general practitioners play a role in facilitating access to specialized health care and in coordinating care from other parts of the health care system. In Sweden, recent marketization efforts in primary health care, particularly *the Patient Choice Reform*, have adversely impacted geographical equity in access health care. This study aimed to examine long-term trends in specialized outpatient health care utilization in the context of the *Patient Choice Reform*, and to do so in regard to demographical, socioeconomic and geographical determinants of health care utilization.

**Method:**

Register data from Region Skåne, the third most populous region in Sweden, was retrieved and a cohort was constructed, describing individuals’ health care utilization between 2007 and 2017. Utilization was measured as the number of outpatient visits to physicians in specialized health care, and based on trajectory analyses trends in utilization were identified. Differences in demographic, geographic and socioeconomic determinants between subgroups with distinct utilization trends were analyzed using logistic regression models.

**Results:**

A closed cohort of 659,298 individuals was constructed. Utilization increased in all sex and age groups except for younger women where utilization decreased. Increased utilization was, in younger individuals, associated with lower socioeconomic status and, in older individuals, with higher socioeconomic status. In all female groups, increased utilization was associated with residence in urban areas and decreased utilization to residence in non-urban areas.

**Conclusion:**

This study provides key insights into long-term trends in outpatient SHC utilization during a time period that overlaps with the *Patient Choice Reform.* The impact of socioeconomic and geographic determinants on utilization varies in magnitude and direction between different age groups of the population in a similar pattern as previously described for primary health care. However, unlike previously reported trends of primary health care utilization, specialized health care utilization in younger women is decreasing.

**Supplementary Information:**

The online version contains supplementary material available at 10.1186/s12913-025-12924-1.

## Background

In Sweden, as in most publicly funded health care systems, equity in health care has a high priority and an aim of the *Swedish Health Care Act* is to ensure equity in access to health care based on need [1]. Affecting equity in health care to various degrees, quasi-markets and public competition have been introduced in health care systems around the world since the 1990 s [2]. Individual liberty in the form of patient choice of health care provider has been a central aspect in many of these reforms [3, 4]. In collectivist health care systems there is, however, a potential conflict between choice and equity, where greater freedom to choose health care providers implies a greater risk of inequalities [5]. Those at higher risk are individuals with little capacity to choose and those who are particularly vulnerable [6].

In Sweden, the state is responsible for the overall health policy, while funding and provision of services are organized by 21 independent regions [7]. The health care system is primarily tax-funded and, to minimize financial barriers to access, citizens are ensured universal coverage with no or minimal patient fees. Less than 1% of total health care expenditure is spent on privately financed health care [8]. As in many other countries, the broader health care system is organized into levels of health care practices with different degrees of specialization and technical sophistication [7]. Half of all doctor visits take place at the primary health care (PHC) level, with a diminishing number of doctor visits as patients get filtered out into higher levels of specialized health care (SHC) [9]. SHC can be defined as health care services requiring medical expertise, equipment or other technologies that cannot be provided in the primary health care setting [10]. With this definition, SHC accounts for about 60% of total health care expenditure [9].

With implications for equity in access to health care, a wave of reforms were initiated throughout Sweden between 2007 and 2010. Despite the insignificant impact of previous marketization efforts [11], these reforms, commonly referred to as the *Patient Choice Reform*, were introduced as a response to dissatisfaction with the performance of public health care services [12]. With the reform, the free establishment of private practices and patient choice of provider was enabled [13]. In Region Skåne, the third most populous region in Sweden, the directives of the reforms were adopted on May 1 st 2009. Initially, the reform concerned PHC exclusively, but in the years following the reform date it gradually became more comprehensive, specifying standards for coordination of elderly care and various diagnose-specific nurse-led clinics, and came to include certain SHC services as well (cataract surgery, 2013; opioid substitution treatment, 2014; ophthalmology, 2014; dermatology, 2014).

Reports of the effects of the *Patient Choice Reform* show changes in PHC provision, affecting both access to and utilization of PHC services. The overall number of PHC centers has increased but new establishments have mainly opened in urban areas or in areas where expected health care needs are lower [14–16]. In urban areas, patient choice has been made more complex due to the multitude and diversification of services offered [17], and demands on patient participation and health literacy are likely to have increased - health literacy refers to individuals’ abilities to access, understand and communicate health-related information needed to make informed decisions [18]. Furthermore, an overall increase in PHC utilization following the reform has been recorded in the general population [14, 19–24], but individuals with higher income or minor symptoms have increased their utilization to a higher degree than those with low income or who are more severely ill [14, 19, 20, 24].

In order to investigate the impact of demographic, geographic and socioeconomic determinants on long-term trends of PHC utilization, we previously performed analyses using a method allowing for person-oriented subgroup analyses [24]. The analyzed time frame overlapped with the implementation of *the Patient Choice Reform*, and the study results demonstrated that the impact of the included determinants on PHC utilization varied in both magnitude and direction across different population groups. However, consistent in all subgroups of becoming high utilizers, was the association with lower socioeconomic status (SES) and residence in urban areas, the latter presumably facilitated by increased PHC accessibility in those areas.

Due to the interdependence between different levels of health care, inequalities in access to PHC can have downstream effects on specialized SHC. Although PHC does not serve as a formal gatekeeper in most regions of the country, general practitioners (GPs) play a key role in facilitating access to SHC and coordinating care across the health care system [7]. With its key functions in disease prevention, early detection, gatekeeping, and care coordination, PHC has significant implications for equity, service utilization, and health care costs [25–28]. In communities with limited access to medical care, hospitalization rates for chronic diseases tend to be higher [29].

Both internationally and in Sweden, equity in access to and utilization of healthcare is known to differ between PHC and SHC. Within the OECD and EU countries, socioeconomic inequalities in access to outpatient health care are described to be relatively lower for PHC than for SHC [30]. Prior to the *Patient Choice Reform* in Sweden, studies have shown a relatively higher utilization of outpatient services in groups with high socioeconomic status (SES) [31–33]. While it has been established that the *Patient Choice Reform* has facilitated an overall increase in PHC utilization, little is known about trends in SHC utilization following the reform, or how observed changes in PHC utilization translate in the SHC setting. Increased geographical inequity of PHC provision and increased demands on patient choice can be regarded as barriers to equity, both in terms of access and utilization. How these PHC trends affect equity in SHC utilization is not known. The purpose of this study was to investigate long-term trends of outpatient SHC utilization in the context of the *Patient Choice Reform*, and to do so in regard to demographical, socioeconomic and geographical determinants of health care utilization.

## Methods

In this study we used the same method as previously presented when investigating trends in PHC utilization [24].

This was a retrospective study, using longitudinal register-based data from Region Skåne to create a closed cohort. Collected data described individual health care utilization to all publicly funded SHC services (both publicly- and privately managed). Privately funded health care services were not included in this study.

Beyond patient-related needs, the utilization of health care services is influenced by factors that predispose individuals to seeking care and factors that enable access to it [34]. In addition to age and sex as need- and predisposing factors, socioeconomic determinants such as income, education, and social status play significant roles in health care utilization and are widely used by Swedish regions as empirical measures for distributing PHC resources [35]. Therefore, at the individual level, data regarding demographic (age and sex) and socioeconomic (income, education and civil status) were obtained from Statistics Sweden and linked to administrative health care utilization data from Region Skåne. Additionally, as the degree of urbanicity in an individual’s community affect service utilization and is relevant in the context of the reform [14, 36, 37], this variable was also included in the analysis.

### Population

A cohort was created of all inhabitants living in Skåne from 2007 to 2017. Individuals aged between 20 and 69 at baseline (1 st of January 2007) were included in the study. Individuals under 20 years of age were excluded to allow the use of income and educational level as a proxy for SES, and individuals over 69 years of age were excluded due to the tapering of the population in older age groups. With a cutoff set at 69 years, the inclusion of retirees was attained. Furthermore, individuals who have had a registered address outside of Skåne at some point during follow-up were excluded from the study.

### Outcome measure

The outcome measure was defined on the individual level as the total number of outpatient visits to physicians in SHC per calendar year. Physician visits solely for research purposes were excluded. Characterization of “SHC” was made on the level of individual clinics. For the purpose of count statistics, relative changes in utilization were compared between periods 2007–2012 and 2012–2017. The breakpoint, set two years after the reform date, was chosen as a midpoint in the follow-up period as a way to distinguish between potential short- and long-term reform effects.

For the purpose of trend analyses, specifically to enable comparisons between paired trajectory groups, four distinct utilization-groups were created based on frequency analyses of the number of annual visits; 0 visits, 1 visit, 2–3 visits, and more than 3 visits. Henceforth, these groups will be denoted low- (0–1 visit), intermediate- (2–3 visits), and high-utilizers (> 3 visits).

### Independent variables

Age, income, educational level, civil status, and municipality of residence were defined at baseline (1 January 2007).

As health care utilization varies across age and sex [38], the analyses were conducted separately for defined groups based on these variables. Additionally, the categorization into age groups served to minimize the effects of age on income. The analyses were made on three different age groups based on age at baseline; 20–34 (young), 35–54 (middle-aged) and 55–69 (older) years of age (corresponding birth years: 1987-73, 1972-53, and 1952-38).

The income variable was defined at the family level as pre-tax family income, adjusted for family size using the concept of consumption units. The family’s total consumption weight was calculated based on a scale that was developed by Statistics Sweden, where the weighting for family members varied depending on their age and relationship to the first adult in the household [39]. for each age group, income was categorized into three equal-sized groups. Pre-tax income encompasses earnings from employment, business, income transfers (e.g., unemployment benefits pension payments, or paid sick leave), and capital gain, but not return of capital.

Educational level was categorized as elementary school, high school or higher education, and civil status to being married/cohabiting or single/divorced/widow/widower.

The degree of urbanicity of municipality of residence was estimated by the municipality group variable. Based on structural parameters such as population and commuting patterns, the Swedish Association of Local Authorities and Regions classifies Swedish municipalities into nine categories [40]. These nine categories were modified to fit into three groups: (1) large cities (population over 200,000) and medium-sized towns (population over 50,000), (2) small towns (population over 15,000) and commuting municipalities near large cities, (3) rural municipalities and commuting municipalities near medium- and small-sized towns. These three groups are for simplicity referred to as urban, semi-urban and rural.

### Statistical method

Separately for each sex and age group, datasets were constructed and analyzed with group-based trajectory modeling (GBTM), a semi-parametric model, GBTM is designed to analyze longitudinal data [41, 42]. By assuming a population composed of a limited mixture of distinct groups defined by their trajectories over time, GBTM can identify subgroups of individuals with similar trajectories [41–43]. With GBTM, the probability of belonging to one subgroup or another (posterior group probability) can be assessed on the individual level. Each individual is assigned to the subgroup to which they have the highest posterior group probability [41, 42]. Subgroups of individuals with similar health care utilization trajectories are here referred to as trajectory groups.

By comparing model fit statistics between nested models, the number of trajectory groups in each dataset was determined. Model fit statistics included evaluation of Bayesian information criterion (BIC) and Akaike’s Information Criterion (AIC), smaller values indicating improvement in model fit. Since the number of observed variables influences the number of trajectory groups, both empirical (improved model fit) and theoretical (model interpretability) aspects were considered when determining the optimal number of trajectory groups in each dataset. Given the size of the study population, statistical power allowed for identification of trajectory groups too small to be useful in clinical- or research work. Hence, only solutions where all identified trajectory groups in a dataset had a prevalence of ≥ 2% of the dataset population were considered for further analyses. The 2% cutoff was arbitrarily chosen.

In the next step, logistic regression was used to describe differences between trajectory groups. To allow for comparison, trajectory groups with similar health care utilization at baseline and with diverging trajectories over time were selected pairwise. Next, pairwise selected trajectory groups were analyzed with logistic regression, using the independent variables age, income, education, civil status, and municipality of residence. In constructed bivariate- and multivariable regression models, all variables were treated as categorical variables except for age which was treated as a continuous variable. Statistical significance was defined at a 5% level and the results were presented as odds ratios (ORs) with 95% confidence intervals (CIs).

Logistic regressions were performed with IBM SPSS version 27 (IBM Corp., Armonk, NY) and GBTMs with SAS 9.4 (SAS Institute, Inc., Cary, NC).

## Results

### Descriptive

In 2007, Skåne had 766,029 residents between aged between 20 and 69, of which 659,298 (86%) were included in the study. Study population demographics are shown in Table 1.

**Table 1:** Cohort population characteristics and general trends in specialized health care utilization in defined sex and age groups between 2007 and 2017

Sex		Male			Female			
Age group (years)		**20–34**	**35–54**	**55–69**	**20–34**	**35–54**	**55–69**	**Total**
Number of individuals (N)		93,427	145,045	88,365	92,405	145,484	94,572	659,298
Income (% of total in age group)	Low income	30.5	31.8	29.8	38.9	36.0	36.6	34,0
	Medium income	30.3	33.4	34.0	35.1	32.7	32.7	33,0
	High income	39.2	34.8	36.2	26.0	31.3	30.7	33,0
Education (% of total in age group)	Primary school	4.9	6.2	16.3	4.0	4.2	17.1	8,2
	Secondary school	39.0	39.6	42.0	35.8	38.9	43.2	39,7
	Higher education	56.1	54.2	41.6	60.3	57.0	39.7	52,2
Civil status (% of total in age group)	Single	81.1	47.0	32.9	72.0	44.3	38.0	51,5
	Married/cohabitant	18.9	53.0	67.1	28.0	55.7	62.0	48,5
Municipality of residence (% of total in age group)	Urban	50.5	40.6	36.1	51.1	40.8	38.0	42,6
	Semi-urban	25.0	30.5	31.9	25.0	30.5	31.4	29,3
	Rural	24.5	28.9	32.0	23.9	28.7	30.5	28,2
Number of annual physician visits per person (mean)	2007	0.76	0.99	1.60	1.59	1.65	2.09	1,44
	2012	0.82	1.12	1.98	1.68	1.65	2.26	1,56
	2017	0.85	1.30	2.61	1.47	1.67	2.58	1,72
Absolute increase in annual physician visits 2007 vs. 2017 (N)		7,203	44,813	89,090	−9,407	2,987	46,289	180,975
Relative increase in annual physician visits 2007 vs. 2017 (%)		11,5	31.3	63.0	−7.3	1.2	23.4	20.7

### Outpatient visits to physicians in specialized health care

In total, 10,914,070 outpatient physician visits were recorded during the study period. Of these visits, 59% were made by women and 7% of the study population made no physician visits at all. From 2007 to 2017 the average number of annual visits per individual rose from 1.44 to 1.72, corresponding to an absolute increase of 180,975 visits or a relative increase of 21% (Table 1). The relative increase in utilization was about the same for the years 2007–2012 as for 2012–2017.

In 2007, all female groups had a distinctly higher average number of annual visits per individual compared to corresponding male groups. Due to a marked increase in utilization in older and middle-aged men, and a decreased utilization in younger women, sex differences in utilization gradually diminished throughout the follow-up period. By 2017, the sex difference in relative utilization between older men and older women had almost disappeared.

Comparing changes in relative utilization between periods 2007–2012 and 2012–2017, both older men and women showed a distinct higher increase in utilization between 2012 and 2017 as compared to 2007–2012. For middle-aged men, middle-aged women and younger men, the increase in utilization was about the same for both periods. In contrast to all other groups, younger women showed a slight increase in utilization in 2007–2012 followed by a decrease in utilization in 2012–2017.

### Trajectories of specialized health care utilization

**Fig. 1:**
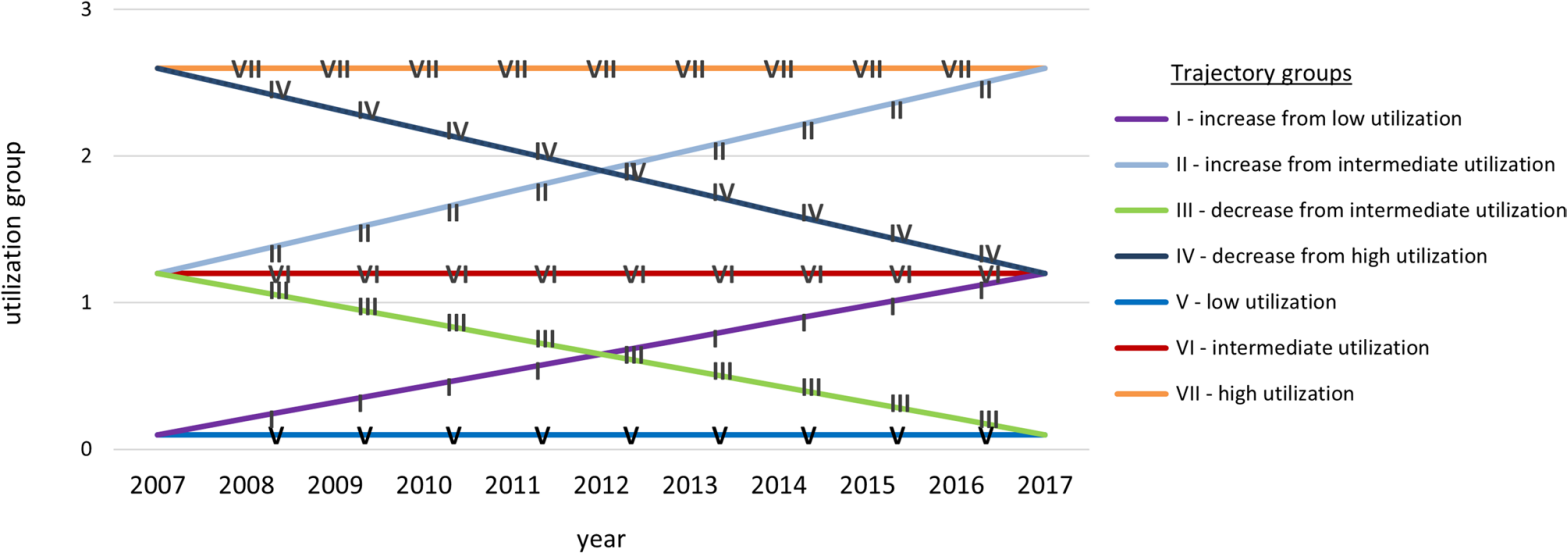
Generic trajectory analysis output. On the Y-axis, the number of annual physician visits in specialized health care per individual are categorized by 4 utilization-groups corresponding to; 0 visits, 1 visit, 2–3 visits, and more than 3 visits. From Kohnke et al. [24]. CC by 4.0 https://creativecommons.org/licenses/by/4.0/. Published 23 November 2023

Following evaluation of model fit statistics (additional Table 1), six datasets of trajectories were chosen for further analysis, one dataset for each defined sex and age group (see additional Fig. 1). Independent variables were recorded to vary between trajectory groups (see additional Tables 2 and 3). As demonstrated in our previous publication [24], datasets displayed a generic pattern of trajectories shown in Fig. 1. Common features in the datasets were the presence of trajectories with relatively unchanged utilization (continuously low-, intermediate-, or high levels of utilization) in combination with trajectories with changing utilization in close to linear fashions. Henceforth, trajectories will be referred to by their Roman numeral as denoted in Fig. 1.

Trajectory groups between low- and intermediate utilization (I, III, V and VI) contained, in general, larger proportions of individuals compared to trajectory groups between intermediate- and high utilization (II, IV and VII). Trajectory groups with changed utilization between low- and intermediate levels were recorded in all sex and age groups and trajectory groups with changed utilization between intermediate- and high levels were recorded in all groups except in younger and middle-aged men. In some datasets, most notably in older men and younger women, trajectories varied from the generic pattern in Fig. 1 by being more dispersed on the y-axis at the starting point.

Common for all datasets, trajectory groups between low utilization and intermediate utilization (I, III, V and VI) contained larger proportions of individuals than those trajectory groups between intermediate and high utilization (II, IV and VII). Trajectory groups with changing utilization between low and intermediate levels were recorded in all sex and age groups, and trajectory groups with changing utilization between intermediate and high levels were recorded in all sex and age groups except in young and middle-aged men.

### Logistic regression

By comparing the target trajectory with a reference trajectory, with both having a similar health care utilization at baseline, changing utilization could be analyzed further by logistic regression. Trajectories serving as references were, in most cases, horizontal, thus indicating an unchanging utilization over time. In some instances, the target trajectory was compared to a reference trajectory with a slope going in a different direction (ex. increase vs. decrease). If not specified otherwise, the reference trajectory used was horizontal.

The results from the bivariable regression model (additional Tables 4 and 5) did not differ considerably from the multivariable model (Tables 2 and 3).

Multivariable regression analyses showed consistent effects of age on utilization. In all datasets, except for younger women, increasing utilization was associated with higher age and decreasing utilization was associated with lower age. The associations between age and utilization were generally stronger in older age groups.

**Table 2:** Multivariable logistic regression on male trajectory groups showing odds ratios (ORs) of change in specialized health care utilization due to differences in predisposing and enabling factors

Age group (years)		20–34		35–54		55–69	
Compared trajectory groups^1^		Group 2 vs. 1 (ref.)	Group 3 vs. 5 (ref.)	Group 3 vs. 2 (ref.)	Group 5 vs. 4 (ref.)	Group 2 vs. 1 (ref.)	Group 3 vs. 5 (ref.)
Corresponding generic trajectory groups^2^		I vs. V (ref.)	III vs. VI (ref.)	I vs. V (ref.)	II vs. III (ref)	I vs. V (ref.)	II vs. III (ref)
Analyzed utilization change		OR for increase from low utilization	OR for decrease from intermediate utilization	OR for increase from low utilization	OR for increase vs. decrease from intermediate utilization	OR for increase from low utilization	OR for increase vs. decrease from intermediate utilization
Age		1.00 (1.00–1.01) ^*^	0.98 (0.97–0.98) ^*^	1.06 (1.05–1.06) ^*^	1.04 (1.04–1.04) ^*^	1.07 (1.07–1.08) ^*^	1.10 (1.09–1.11) ^*^
Income	Low income	1	1	1	1	1	1
	Middle income	0.99 (0.94–1.04) ^*^	1.19 (1.12–1.26) ^*^	0.94 (0.91–0.98) ^*^	0.82 (0.78–0.86) ^*^	1.18 (1.12–1.24) ^*^	1.04 (0.96–1.13) ^*^
	High income	0.90 (0.86–0.94) ^*^	1.35 (1.27–1.43) ^*^	0.91 (0.87–0.95) ^*^	0.81 (0.77–0.85) ^*^	1.35 (1.27–1.42) ^*^	1.26 (1.16–1.36) ^*^
Education	Primary school	1	1	1	1	1	1
	Secondary school	0.76 (0.70–0.84) ^*^	1.31 (1.18–1.44) ^*^	0.96 (0.89–1.03) ^*^	0.85 (0.78–0.92) ^*^	1.08 (1.02–1.15) ^*^	1.09 (0.99–1.19) ^*^
	Higher education	0.63 (0.58–0.70) ^*^	1.63 (1.48–1.80) ^*^	0.91 (0.85–0.98) ^*^	0.74 (0.68–0.80) ^*^	1.14 (1.07–1.22) ^*^	1.23 (1.12–1.35) ^*^
Civil status	Single	1	1	1	1	1	1
	Married or cohabitant	1.05 (1.00–1.10) ^*^	1.12 (1.05–1.19) ^*^	1.00 (0.97–1.03) ^*^	0.95 (0.91–0.99) ^*^	1.15 (1.10–1.20) ^*^	1.07 (1.00–1.15) ^*^
Municipality group	Urban	1	1	1	1	1	1
	Semi-urban	0.99 (0.94–1.04) ^*^	0.97 (0.92–1.02) ^*^	0.97 (0.94–1.01) ^*^	0.94 (0.90–0.99) ^*^	1.04 (0.99–1.09) ^*^	0.90 (0.83–0.97) ^*^
	Rural	0.92 (0.88–0.97) ^*^	1 0.00 (0.94–1.05) ^*^	1 0.00 (0.96–1.04) ^*^	0.96 (0.92–1.01) ^*^	1.01 (0.96–1.06) ^*^	0.90 (0.83–0.97) ^*^

^1^ as shown in supplementary information

^2^ as shown in Fig. 1

^*^P<0,05

**Table 3:** Multivariable logistic regression on female trajectory groups showing odds ratios (ORs) of change in specialized health care utilization due to differences in predisposing and enabling factors

Age group (years)		20–34			35–54				55–69		
Compared trajectory groups^1^		Group 5 vs. 1 (ref.)	Group 4 vs. 3 (ref.)	Group 4 vs. 2 (ref.)	Group 4 vs. 1 (ref.)	Group 3 vs. 5 (ref.)	Group 3 vs. 2 (ref.)	Group 6 vs. 7 (ref.)	Group 2 vs. 1 (ref.)	Group 3 vs. 4 (ref.)	Group 5 vs. 6 (ref.)
Corresponding generic trajectory groups^2^		I vs. V (ref.)	II vs. III (ref)	II vs. III (ref)	I vs. V (ref.)	II vs. III (ref)	II vs. III (ref)	IV vs. VII (ref.)	I vs. V (ref.)	II vs. III (ref)	IV vs. VII (ref.)
Analyzed utilization change		OR for increase from low utilization	OR for increase vs. decrease from intermediate utilization	OR for increase vs. decrease from intermediate utilization	OR for increase from low utilization	OR for increase vs. decrease from intermediate utilization	OR for increase vs. decrease from intermediate utilization	OR for decrease from high utilization	OR for increase from low utilization	OR for increase vs. decrease from intermediate utilization	OR for decrease from high utilization
Age		0,97 (0,96 − 0,97) ^*^	0,97 (0,96 − 0,98) ^*^	0,97 (0,96 − 0,98) ^*^	1,02 (1,02 − 1,02) ^*^	1,05 (1,04 − 1,05) ^*^	1,02 (1,01–1,02)	0,99 (0,98 − 0,99) ^*^	1,06 (1,05 − 1,06) ^*^	1,05 (1,05 − 1,06) ^*^	0,97 (0,96 − 0,98) ^*^
Income	Low income	1	1	1	1	1	1	1	1	1	1
	Middle income	0,98 (0,93 − 1,02) ^*^	0,74 (0,69 − 0,80) ^*^	0,83 (0,77 − 0,89) ^*^	1,02 (0,98 − 1,07) ^*^	0,78 (0,74 − 0,82) ^*^	0,83 (0,79 − 0,88)	1,05 (0,96 − 1,14) ^*^	1,13 (1,07 − 1,20) ^*^	1,04 (0,99 − 1,09) ^*^	0,99 (0,92 − 1,07) ^*^
	High income	0,99 (0,94 − 1,04) ^*^	0,67 (0,62 − 0,73) ^*^	0,76 (0,69 − 0,83) ^*^	1,07 (1,02 − 1,11) ^*^	0,79 (0,74 − 0,83) ^*^	0,80 (0,75 − 0,84)	1,17 (1,06 − 1,28) ^*^	1,21 (1,13 − 1,29) ^*^	1,11 (1,05 − 1,17) ^*^	1,04 (0,95 − 1,13) ^*^
Education	Primary school	1	1	1	1	1	1	1	1	1	1
	Secondary school	0,87 (0,78 − 0,98) ^*^	0,72 (0,62 − 0,83) ^*^	0,96 (0,83 − 1,10) ^*^	1,12 (1,02 − 1,22) ^*^	0,95 (0,85 − 1,07) ^*^	0,97 (0,87 − 1,08)	1,07 (0,93 − 1,24) ^*^	1,08 (1,02 − 1,15) ^*^	1,02 (0,96 − 1,08) ^*^	0,90 (0,83 − 0,90) ^*^
	Higher education	0,82 (0,73 − 0,92) ^*^	0,55 (0,48 − 0,64) ^*^	0,90 (0,78 − 1,04) ^*^	1,09 (1,00–1,19) ^*^	0,79 (0,71 − 0,88) ^*^	0,85 (0,77 − 0,95)	1,16 (1,01–1,35) ^*^	1,13 (1,06 − 1,21) ^*^	1,05 (0,99 − 1,11) ^*^	0,91 (0,83 − 1,00) ^*^
Civil status	Single	1	1	1	1	1	1	1	1	1	1
	Married or cohabitant	0,89 (0,85 − 0,93) ^*^	0,71 (0,65 − 0,76) ^*^	0,77 (0,71 − 0,84) ^*^	1,07 (1,03 − 1,11) ^*^	0,97 (0,92 − 1,02) ^*^	0,95 (0,91 − 1,00)	1,03 (0,96 − 1,11) ^*^	1,02 (0,97 − 1,07) ^*^	0,99 (0,95 − 1,03) ^*^	1,04 (0,97 − 1,11) ^*^
Municipality group	Urban	1	1	1	1	1	1	1	1	1	1
	Semi-urban	0,81 (0,77 − 0,85) ^*^	0,87 (0,81 − 0,95) ^*^	0,95 (0,88 − 1,03) ^*^	0,88 (0,84 − 0,91) ^*^	0,83 (0,79 − 0,88) ^*^	0,95 (0,91 − 1,00)	1,22 (1,12 − 1,32) ^*^	1,00 (0,94 − 1,05) ^*^	0,99 (0,94 − 1,03) ^*^	1,21 (1,13 − 1,30) ^*^
	Rural	0,82 (0,78 − 0,86) ^*^	0,81 (0,75 − 0,88) ^*^	0,96 (0,88 − 1,04) ^*^	0,83 (0,80 − 0,87) ^*^	0,83 (0,78 − 0,87) ^*^	0,93 (0,88 − 0,98)	1,28 (1,17 − 1,40) ^*^	0,99 (0,93 − 1,04) ^*^	0,99 (0,95 − 1,04) ^*^	1,29 (1,20 − 1,40) ^*^

^1^ as shown in supplementary information

^2^ as shown in Fig. 1

^*^P<0,05

### Logistic regression – increasing utilization from low levels

Analyses of the effect of income and education on increased utilization from low- to intermediate levels (I) showed that these variables affect utilization in different directions in older age groups as compared to younger age groups. Among older men, the OR for increased utilization from low levels (I) was 1.35 (CI 1.27–1.42) for high vs. low income and 1.14 (CI 1.07–1.22) for higher education vs. primary school. Corresponding numbers among older women were 1.21 (CI 1.13–1.29) for high vs. low income and 1.13 (CI 1.06–1.21) for higher education vs. primary school. Among middle-aged women, the association between increased utilization (I) and income or education was in the same direction as in older men and women but was weaker. In contrast, increased utilization (I) in middle-aged men, younger men, and younger women was associated with lower income or lower education. Within these three groups, the strongest association was recorded among younger men where the OR for increased utilization (I) was 0.90 (CI 0.86–0.94) for high vs. low income and 0.63 (CI 0.58–0.70) for higher education vs. primary school. Among younger men, utilization change in the opposite direction, from intermediate to low levels (III), was associated with high income and higher education. Here, OR for decreased utilization (III) was 1.35 (CI 1.27–1.43) for high vs. low income and 1.63 (CI 1.48-1-80) for higher education vs. primary school.

### Logistic regression – increasing vs. decreasing utilization from intermediate levels

Trajectories with changing utilization from the intermediate level (II and III) were more complex to analyze due to the positioning and slopes of reference trajectories. At this level, the association between socioeconomic variables and changed utilization was analyzed by comparing trajectories of increasing utilization (II) with trajectories of decreasing utilization (III), the latter serving as reference.

The effect of income and education on increased utilization from intermediate levels (II) was distinctly different in the oldest age group as compared to middle-aged and younger age groups. When compared to decreasing utilization (III), increasing utilization from intermediate levels (II) was, among older men and older women, associated with higher income. For older men, the OR for increased utilization (II) was 1.26 (CI 1.16-1-36) for high vs. low income, and for older women it was 1.11 (CI 1.05–1.17). In contrast, the corresponding data for middle-aged men, middle-aged women and younger women were associated with lower income and lower education. Recorded ORs for increased utilization for individuals with high vs. low income (II) were 0.81 (CI 0.77–0.85) for middle-aged men, 0.79 (CI 0.74–0.83), 0.80 (CI 0.75–0.84) for middle-aged women and 0.67 (CI 0.63–0.73) for young women. The two recorded ORs for middle-aged women correspond to the comparison of the target trajectory with two different reference trajectories. Similar to the association with income, recorded ORs for increased utilization (II) for individuals with higher education vs. primary school were 0.74 (CI 0.68–0.80) for middle-aged men, 0.79 (CI 0.71–0.78) for middle-aged women and 0.55 (CI 0.48–0.64) for young women.

### Logistic regression – decreasing utilization from high levels

Trajectories of decreasing utilization from high levels (IV) were recorded for older and middle-aged women. Analyzes of these changes showed no clear association with socioeconomic variables.

### Logistic regression – civil status

As an additional indicator of socioeconomic position, civil status was evaluated in terms of association with changed utilization. The effect of being married/cohabitant on change in utilization was similar to the effect of higher income and higher educational level in all age groups except for older women. Given the magnitude of the ORs, income or education rather than civil status had a greater effect on change in utilization.

### Logistic regression – municipality of residence

The effect of municipality of residence on utilization was analyzed regarding the municipality’s level of urbanicity. Differences in the effect on change in utilization were small between residence in semi-urban and rural areas when these were compared to residence in urban areas. For younger and middle-aged women, increasing utilization from both low (I) and intermediate (II) levels were associated with residence in urban areas. In all female groups, decreasing utilization (both III and IV) was associated with residence in non-urban areas. Also in older men, increasing utilization (II) was associated with residence in urban areas, but compared to female groups the association was weaker. Except for older men, no clear pattern of associations between utilization and urbanicity could be observed in male groups.

## Discussion

Following the *Patient Choice Reform* of Swedish PHC, an overall increase in PHC utilization has been recorded in the general population. The increase has been shown to be unevenly distributed between individuals of different SES and as a result of geographical determinants [14–16]. To provide a more comprehensive understanding of trends in health care utilization in context of the reform, this follow-up examines long-term trends in outpatient SHC utilization. First, our findings indicate a steady increase in SHC utilization in the general population, including all sex and age groups, except in younger women where utilization was shown to decrease. Second, socioeconomic determinants impact utilization in different directions across distinct subgroups of the population. Increased utilization among younger individuals is associated with lower SES, whereas in older individuals it is associated with higher SES. Third, in female groups, there is a clear relationship between the direction of utilization change and municipality of residence. Increased utilization among middle-aged and younger women is associated with residence in urban areas, while decreased utilization across all female groups is associated with residence in non-urban areas.

To our knowledge, this study is the first to evaluate trends in SHC utilization overlapping the *Patient Choice Reform*. The result of this study should be contextualized by previous findings of the effect of the reform on PHC accessibility and utilization. In a previous study on the same population, overall PHC utilization was recorded to markedly increase between 2007 and 2012 and then to be more static between 2012 and 2017 [24]. In contrast, this study shows a steady increase in outpatient SHC utilization between 2007 and 2017. Followingly, the accentuated increase as observed in PHC utilization between 2007 and 2012 does not seem to have translated to SHC.

At the group level, changes in outpatient SHC utilization differed between sex and age groups. In all age groups, women had a comparable higher relative utilization at starting point than men, and in all age groups sex differences in utilization decreased throughout follow-up. In middle-aged and older age groups, this does not necessarily imply a more gender-equitable health care but is rather an expression of heterogeneity in the effect of age on health care needs between men and women. The life expectancy for 65-year-old men in Malmö is 2.8 years shorter than that of women the same age [44] and, hence, SHC utilization in middle-aged and older men is expected to increase more than in age-matched women.

Standing out from the general pattern of increased outpatient SHC utilization during follow-up, utilization in younger women was observed to decrease. Comparing these results with previous findings of PHC utilization [24], the relative increase in SHC utilization in younger men is of the same magnitude as their increase in PHC utilization during the same time period. However, for younger women, the decrease in SHC utilization contrasts with the previously shown marked increase in PHC utilization. For younger women, this suggests a shift in health care utilization between levels of the health care system, where they, to a larger degree, utilize PHC instead of SHC. For younger women, the effect of increased PHC accessibility following the reform might have resulted in that they, to a higher degree, get their health care needs met within PHC, and consequently utilize SHC to a lesser degree. Utilization change on one level does not, however, need to be linked to a change on another level, and observed divergent trends in PHC and SHC utilization in younger women can be two isolated events, each with their own underlying causes. Decreased SHC accessibility for specific patient groups, affecting younger women to a higher degree than other sex and age groups, could be an underlying cause of the decrease in SHC utilization in younger women. To the knowledge of the authors, no such changes have been formally expressed from Region Skåne or elsewhere. Still, it cannot be excluded that minor changes in referral practices, or changes in range of services provided by specific SHC clinics, could have affected utilization.

Published data on the effect of SES on SHC utilization in the Swedish setting is sparse. In a study published prior to the Patient Choice Reform, higher-income groups were recorded to utilize outpatient health care services and privately managed SHC to a higher degree than low-income groups [33]. Our data suggest socioeconomic determinants have distinctly different effects on utilization in different subgroups of the population. Following the same pattern as previously presented regarding PHC utilization [24], increased SHC utilization in younger individuals is associated with lower SES, while similar changes in older individuals are associated with higher SES. To the best of our knowledge, the reason for this difference across age groups is not known. A possible explanation might reside in how the choice reform has increased the complexity of the health care system and thus increased demands on patient participation and individuals’ health literacy. However, depending on subgroup health literacy characteristics and the accessibility of health care for these specific subgroups, it is conceivable that increased health care complexity can drive utilization in the same direction in two seemingly distinct groups. If this were true, the choice reform could be an underlying cause of the observed pattern of increased utilization in both younger women and older men. This idea remains speculative and the mechanism through which SES affects utilization in the Swedish patient choice model needs further exploration.

With the *Patient Choice Reform*, free establishment of private practices was enabled and the number of PHC providers increased promptly after the reform date. With new establishments mainly opening in urban areas or in areas where expected healthcare needs were lower [14–16], geographical inequity in PHC provision increased. To the knowledge of the authors, there have been no reports on trends in SHC utilization following the reform. In this study, increased utilization in younger and middle-aged women is associated with residence in urban areas, with the association being stronger in the younger age group. Similarly, all female groups with decreased utilization are associated with residence in non-urban areas. A similar pattern has been recorded regarding changes in PHC utilization following the reform [24]. Higher health care utilization, by means of increased accessibility, is expected for individuals living in urban areas, but why this association is predominantly observed in female groups is unclear. Given the similarity between PHC and SHC data, increased utilization in women with residence in urban areas might be an expression of changed health care seeking behavior in female urban population groups, plausibly facilitated by improved health care accessibility in urban areas.

The strength of this study is the access to reliable data sources and that we have studied a large, constant population over a ten-year period. The data in this study are based on register information collected for administrative purposes. In Sweden, there is a long tradition of recording, storing, and managing information in registers. This tradition entails both individual regions and government authorities and data validity in these registers is known to be high.

Potentially limiting the interpretations of the results is that advancements in medical care over the ten-year observation period may have contributed to an increase in outpatient SHC utilization. As medical technologies and treatment protocols evolve, certain procedures and treatments that were previously conducted in inpatient settings may have shifted to outpatient care. Such a trend could partially explain the observed increase in outpatient visits, particularly in the later years of the study. While this reflects improvements in efficiency and accessibility, it also highlights the need for cautious interpretation of utilization trends, as changes in health care delivery models may influence long-term patterns of care.

The closed cohort design of the study entails both advantages as well as disadvantages in terms of data analysis. With a closed cohort design, it is possible to follow individuals over time in a way that would not be possible with an ecological design. The closed cohort design does not allow for new entries or exits during the follow-up and potential confounders due to fluctuations in the population can thus be disregarded. Still, beyond changes in health care organization, utilization on the population level can be affected by a range of different factors including the external environment (physical, political, and environmental) and population characteristics (attitudes, predisposing characteristics, enabling resources and need) [34]. External factors have remained stable throughout the study period and are not believed to have influenced the results. Population characteristics such as education, income, and health care need, however, are likely to have changed during the 10-year follow-up. Since early adulthood and retirement are time points where education and income typically change, we performed sensitivity analyses defining income- and educational level at the endpoint instead of at baseline. These sensitivity analyses showed little or no effects on outcomes.

Due to the closed cohort design of the study, a gradual increase in utilization over time in the general population is expected. However, increasing age and attrition are likely to impact subgroups of the population in varying degrees and identified trajectory groups should thus be regarded as a representation of the heterogeneity in utilization affected by changes in health care organization, as well as heterogeneity in changed health care seeking behavior and health care needs. Considering the appropriation of health care services according to health care needs as the basis for equitable health, the absence of control for morbidity limits this study. Adjusted Clinical Groups (ACG), developed by The Johns Hopkins University to evaluate the relationship between individual morbidity and utilization [45], was applied as an independent variable in an initial regression model. In this model, all individuals were subscribed one of six Resource Utilization Bands (RUB) – RUB 0 corresponding to no need for health care and RUB 5 a high need, each RUB consisting of individuals with the same type and degree of comorbidity. Regardless of defining RUB at baseline or endpoint, ORs for RUB showed an absolute correlation to health care utilization, adding no useful information. Thus, ACG was excluded from the final regression model.

Other limitations of this study concern potential loss of information and degree of uncertainty in analyses due to inherent characteristics of the study method. According to the method used here, a premise for analyzing differences in utilization trajectories is the categorization of the outcome variable into utilization-groups based on the annual number of physician visits. The categorization implies a loss of information that theoretically can affect the sensitivity of the results negatively. Due to the large study population, and the large amount of SHC utilization data, we do not believe this loss of information to be sufficient to affect the final results. Furthermore, when running the trajectory datasets, no lower limit in posterior probability was applied, and each individual was assigned to the subgroup to which they had the highest posterior group probability. Theoretically, the posterior probability could be low for all the available subgroups and hence introduce uncertainty that would carry forward in the next step of the analysis - the regression models. To evaluate potential error, a sensitivity analysis was carried out where the degree of uncertainty in the trajectory model was limited by disregarding data points with a posterior probability below 0.7. The results from this sensitivity analysis did not vary considerably compared to the original model, and concerns regarding error due to an increased degree of uncertainty were disregarded.

With some considerations, the results of this study can be generalized to other Swedish regions. Foremost, these considerations concern population demographics and regional variation in health care provision. In addition, differences in regional choice reform models need to be taken into account, since these are known to differ in terms of comprehensiveness and reimbursement model. For the same reasons stated above, generalizing these results to patient choice reforms in non-Swedish settings should be done with caution, as the degree of variation in the aforementioned factors is likely to be even greater.

## Conclusions

In Sweden, as in many other countries, marketization and increased patient choice are reshaping health care delivery. A key example is the *Patient Choice Reform* in PHC. Through addressing key health risks in the population, PHC influences overall health system efficiency. While the reform’s impact on PHC provision and utilization are well studied, interlevel health care effects, however, remain largely unexplored. In this context, this study offers valuable insights into long-term trends in outpatient SHC utilization during a period that overlaps with the reform.

In this study, increased SHC utilization was recorded in all sex and age groups except that for younger women who showed decreased utilization. The impact of socioeconomic and geographic determinants on SHC utilization varied in magnitude and direction between groups of the population, following a pattern previously described for PHC. Increased utilization in younger individuals was associated with lower SES, while a similar change in older individuals was associated with higher SES. Furthermore, increased utilization in younger and middle-aged women was associated with residence in urban areas while all female groups with decreased utilization were associated with residence in non-urban areas.

It’s likely there are several plausible explanations behind observed group differences in SHC utilization. In older parts of the population, differences in health care needs between groups are likely a major contributing factor while in the younger part of the population, changed health care-seeking behavior and increased importance of health literacy in the patient choice system could contribute as well. To get a more complete understanding of the impact of the reform on equity in utilization, future studies should include continuity of care as a quality aspect of utilization. Furthermore, the mechanism through which SES affects utilization in the Swedish patient choice model is unclear and needs further exploration.

## Supplementary Information

Supplementary Material 1: Additional Fig. 1. Trajectory analysis datasets. Trajectory analysis output showing trajectories of specialized health care utilization between 2007 and 2017. On the Y-axis, the number of annual physician visits in specialized health care per individual are categorized by 4 utilization-groups corresponding to; 0 visits, 1 visit, 2–3 visits, and more than 3 visits

Supplementary Material 2: Additional Table 1. Trajectory fit statistics. Additional Table 2. Trajectory group characteristics at baseline for males. Numbers indicate percent (%) of subgroup total if not otherwise specified. Additional Table 3. Trajectory group characteristics at baseline for females. Numbers indicate percent (%) of subgroup total if not otherwise specified. Additional Table 4. Bivariate logistic regression on male trajectory groups showing odds ratios (ORs) of change in SHC utilization due to differences in predisposing and enabling factors. Additional Table 5. Bivariate logistic regression on female trajectory groups showing odds ratios (ORs) of change in SHC utilization due to differences in predisposing and enabling factors

## Data Availability

The datasets generated and analyzed during this study are available from the corresponding author upon reasonable request.

## Abbreviations

GP: General practitioner
PHC: Primary health care
SES: Socioeconomic status
SHC: Specialized health care
