# Supplementary figures and images for: Long-term trends in specialized outpatient health care utilization: an analysis in the context of a primary health care reform
Source: BMC Health Serv Res. 2025 Jul 1;25:879. doi: 10.1186/s12913-025-12924-1 (PMC12220664; doi:10.1186/s12913-025-12924-1)

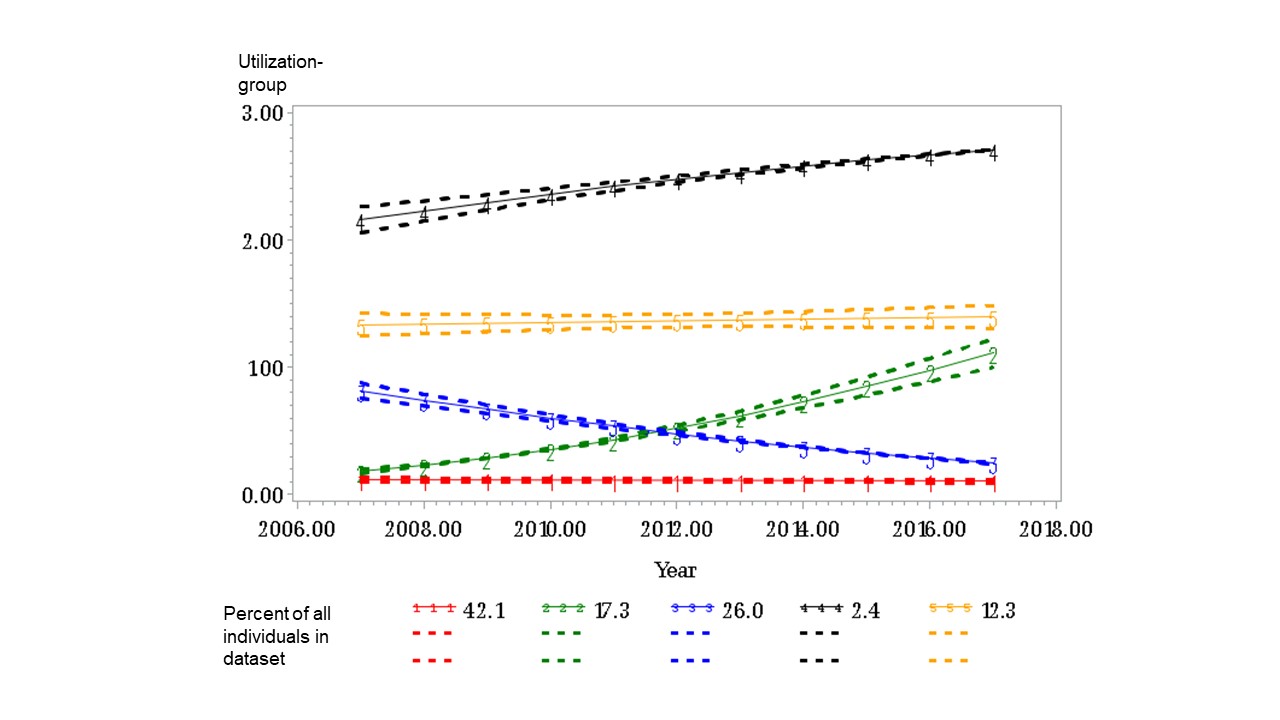

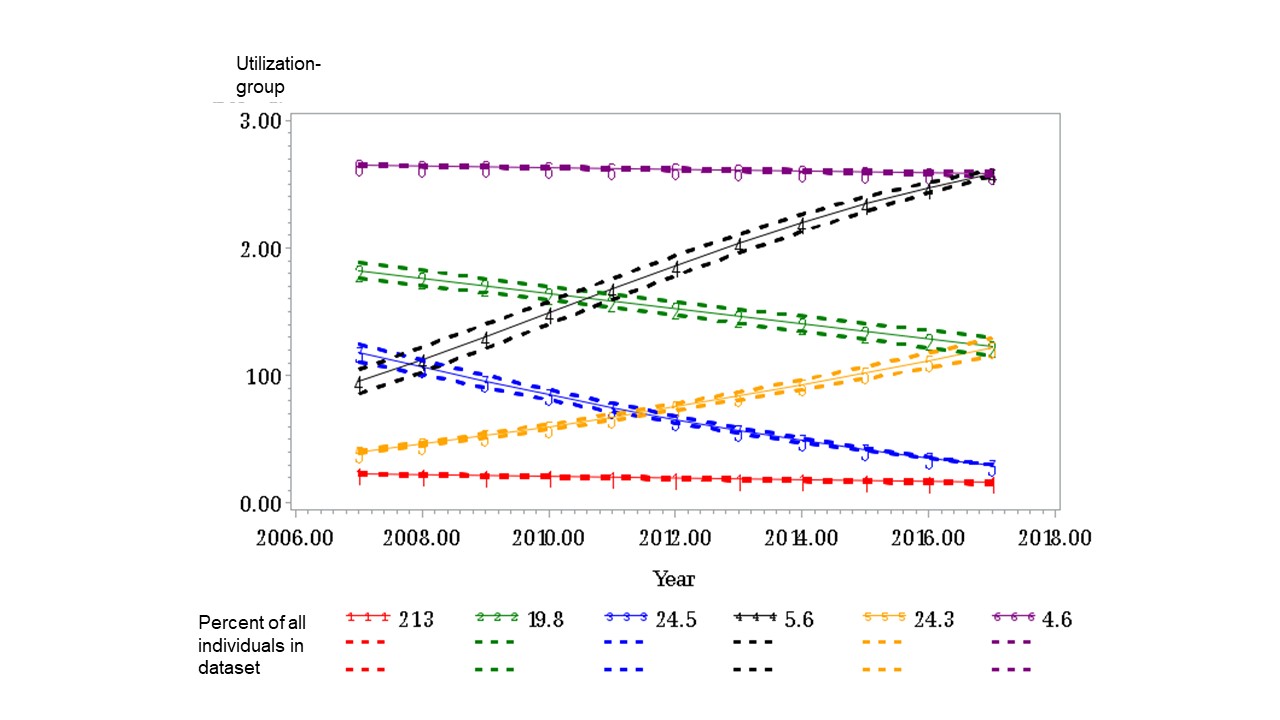

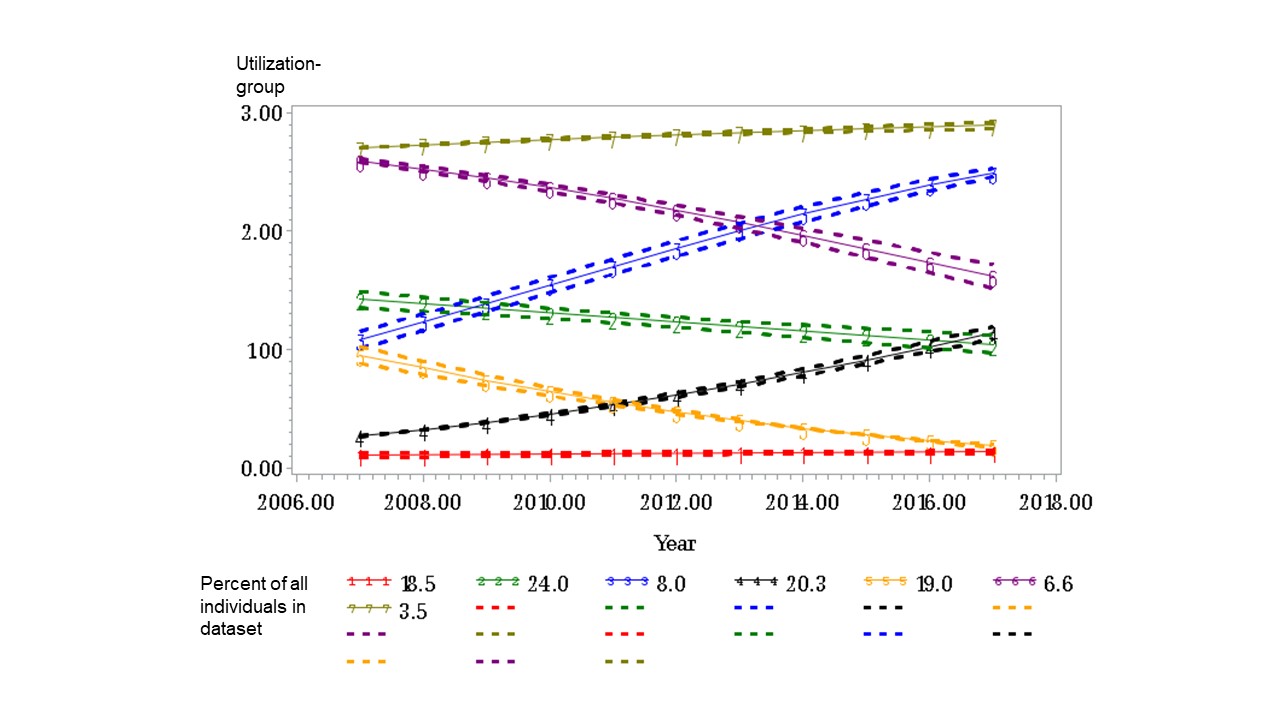

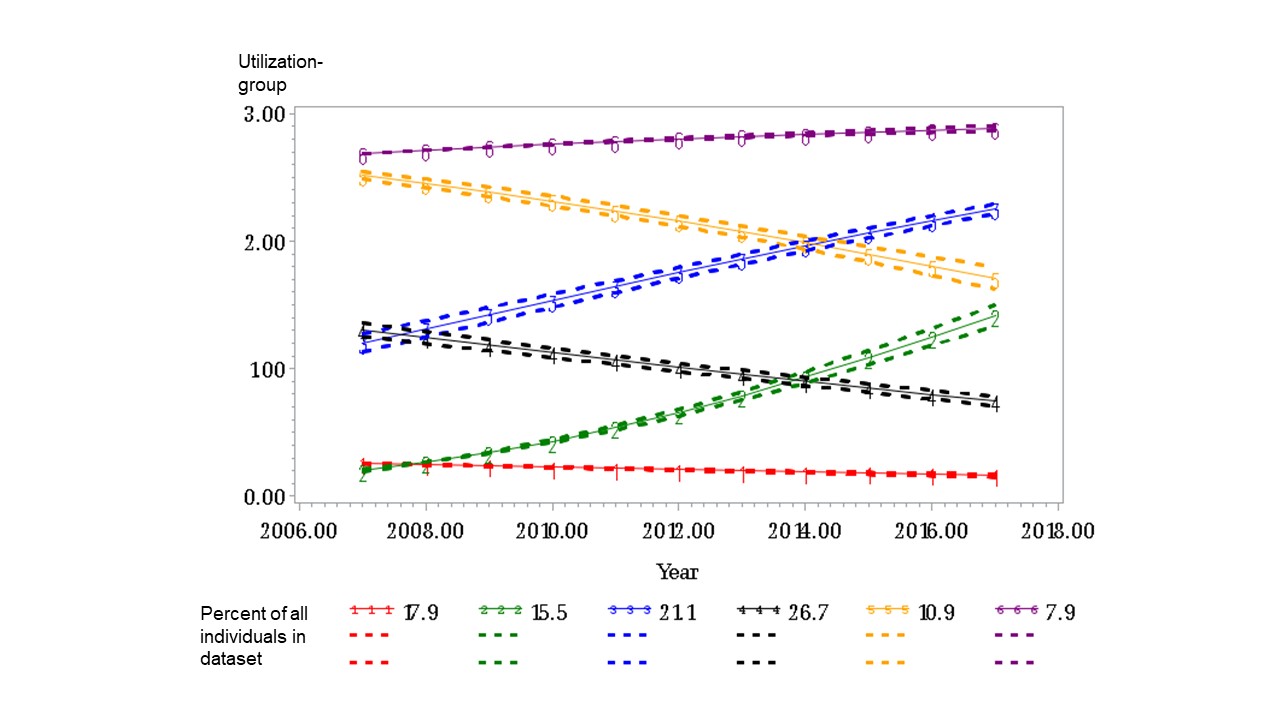

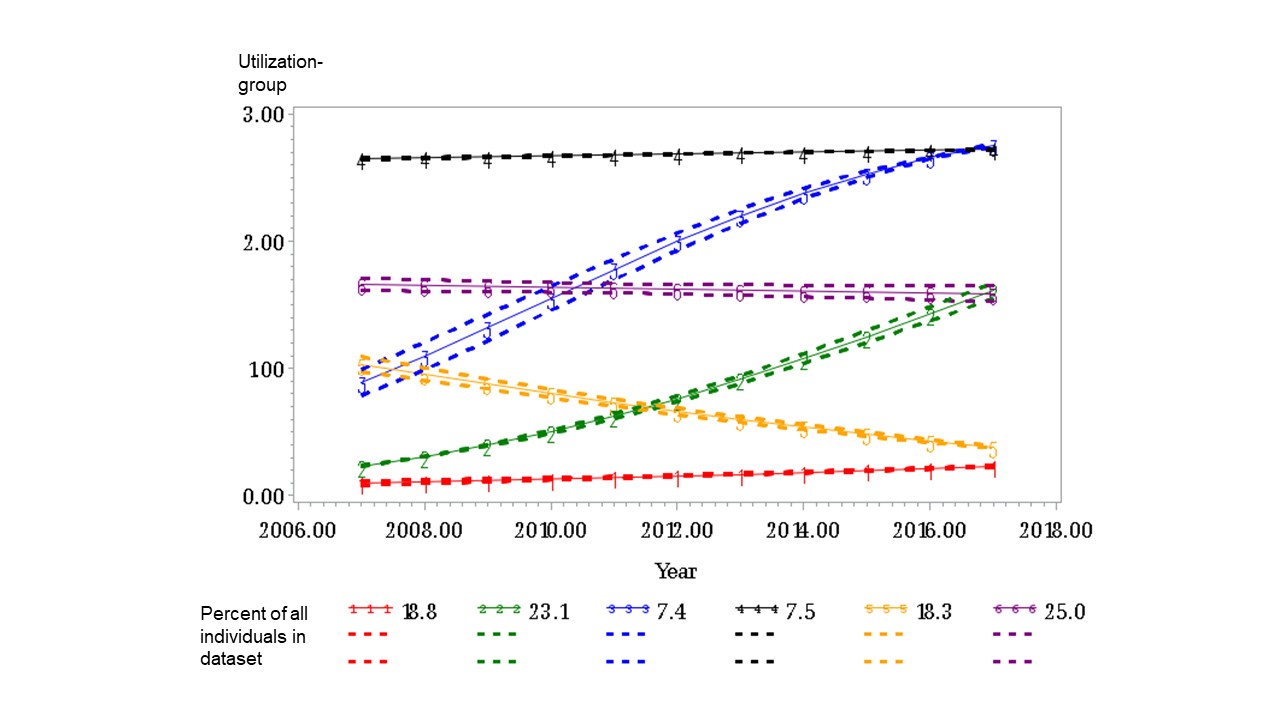

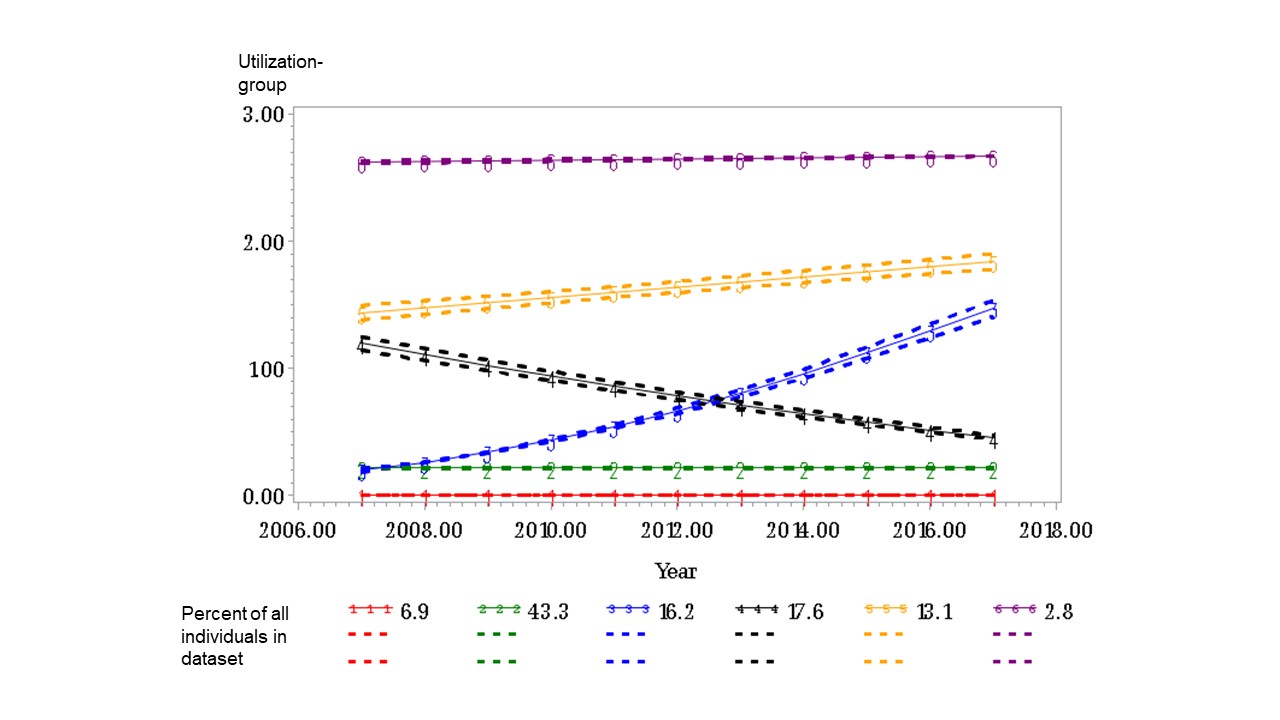


**b**. Women 55-69 years

**a**. Men 55-69 years

**d**. Women 35-54 years

**c**. Men 35-54 years

**f**. Women 20-34 years

**e**. Men 20-34 years

Supplement: Supplementary file 1 — Supplementary Material 1: Additional Fig. 1. Trajectory analysis datasets. Trajectory analysis output showing trajectories of specialized health care utilization between 2007 and 2017. On the Y-axis, the number of annual physician visits in specialized health care per individual are categorized by 4 utilization-groups corresponding to; 0 visits, 1 visit, 2–3 visits, and more than 3 visits [file 12913_2025_12924_MOESM1_ESM.docx]
