# Supplementary material for: Long-term trends in specialized outpatient health care utilization: an analysis in the context of a primary health care reform
Source: BMC Health Serv Res. 2025 Jul 1;25:879. doi: 10.1186/s12913-025-12924-1 (PMC12220664; doi:10.1186/s12913-025-12924-1)
Supplement: Supplementary file 2 — Supplementary Material 2: Additional Table 1. Trajectory fit statistics. Additional Table 2. Trajectory group characteristics at baseline for males. Numbers indicate percent (%) of subgroup total if not otherwise specified. Additional Table 3. Trajectory group characteristics at baseline for females. Numbers indicate percent (%) of subgroup total if not otherwise specified. Additional Table 4. Bivariate logistic regression on male trajectory groups showing odds ratios (ORs) of change in SHC utilization due to differences in predisposing and enabling factors. Additional Table 5. Bivariate logistic regression on female trajectory groups showing odds ratios (ORs) of change in SHC utilization due to differences in predisposing and enabling factors [file 12913_2025_12924_MOESM2_ESM.docx]

**Additional Table 1**. Trajectory fit statistics.

| Sex | Age-group (years) | Number of trajectories | Number of  datapoints | BIC | AIC | Log-likelihood ratio |
| --- | --- | --- | --- | --- | --- | --- |
| Males | **20–34** | 3 | 1,027,697 | -979,274 | -979,221 | -979,212 |
|  |  | 4 | 1,027,697 | -976,207 | -976,136 | -976,124 |
|  |  | 5* | 1,027,697 | -972,657 | -972,568 | -972,553 |
|  |  | 6 | 1,027,697 | -971,808 | -971,701 | -971,683 |
|  | **35–54** | 3 | 1,595,473 | -1,690,229 | -1,690,174 | -1,690,165 |
|  |  | 4 | 1,595,473 | -1,683,595 | -1,683,521 | -1,683,509 |
|  |  | 5 | 1,595,473 | -1,678,835 | -1,678,743 | -1,678,728 |
|  |  | 6* | 1,595,473 | -1,674,010 | -1,673,899 | -1,673,881 |
|  |  | 7 | 1,595,473 | -1,671,769 | -1,671,640 | -1,671,619 |
|  | **55–69** | 3 | 972,015 | -1,269,715 | -1,269,662 | -1,269,653 |
|  |  | 4 | 972,015 | -1,263,338 | -1,263,267 | -1,263,255 |
|  |  | 5 | 972,015 | -1,255,814 | -1,255,725 | -1,255,710 |
|  |  | 6* | 972,015 | -1,253,264 | -1,253,158 | -1,253,140 |
|  |  | 7 | 972,015 | -1,253,965 | -1,253,841 | -1,253,820 |
| Females | **20–34** | 3 | 1,016,455 | -1,303,278 | -1,303,225 | -1,303,216 |
|  |  | 4 | 1,016,455 | -1,299,656 | -1,299,585 | -1,299,573 |
|  |  | 5 | 1,016,455 | -1,294,499 | -1,294,411 | -1,294,396 |
|  |  | 6* | 1,016,455 | -1,292,479 | -1,292,372 | -1,292,354 |
|  | **35–54** | 3 | 1,600,313 | -2,015,330 | -2,015,275 | -2,015,266 |
|  |  | 4 | 1,600,313 | -2,005,688 | -2,005,615 | -2,005,603 |
|  |  | 5 | 1,600,313 | -1,997,421 | -1,997,329 | -1,997,314 |
|  |  | 6 | 1,600,313 | -1,993,428 | -1,993,317 | -1,993,299 |
|  |  | 7* | 1,600,313 | -1,990,365 | -1,990,236 | -1,990,215 |
|  | **55–69** | 3 | 1,040,303 | -1,401,622 | -1,401,568 | -1,401,559 |
|  |  | 4 | 1,040,303 | -1,393,725 | -1,393,654 | -1,393,642 |
|  |  | 5 | 1,040,303 | -1,387,644 | -1,387,555 | -1,387,540 |
|  |  | 6* | 1,040,303 | -1,384,596 | -1,384,489 | -1,384,471 |
|  |  | 7 | 1,040,303 | -1,385,052 | -1,384,927 | -1,384,906 |
| AIC = Akaike information criterion BIC =Bayesian information criterion  * denotes model chosen for further analyses | | | | | | |

**Additional Table 2**. Trajectory group characteristics at baseline for males. Numbers indicate percent (%) of subgroup total if not otherwise specified.

| Age group (years) | | 20–34 | | | | | 35–54 | | | | | | | 55–69 | | | | | |
| --- | --- | --- | --- | --- | --- | --- | --- | --- | --- | --- | --- | --- | --- | --- | --- | --- | --- | --- | --- |
| Trajectory group | | **Group 1** | **Group 2** | **Group 3** | **Group 4** | **Group 5** | **Group 1** | **Group 2** | **Group 3** | **Group 4** | **Group 5** | **Group 6** | **Group 1** | | **Group 2** | **Group 3** | **Group 4** | **Group 5** | **Group 6** |
| Age (mean) | | 27.4 | 27.4 | 27.2 | 27.8 | 27.5 | 43.2 | 43.5 | 45.1 | 44.4 | 45.6 | 45.9 | 60.3 | | 61.3 | 62.3 | 62.6 | 60.9 | 61.9 |
| Income (% of subgroup total) | 40.6 | 37.6 | 40.9 | 25.0 | 35.2 | 34.2 | 34.1 | 36.0 | 36.6 | 34.2 | 32.6 | 24.9 | 32.8 | | 37.7 | 39.2 | 36.6 | 34.2 | 37.8 |
|  | 29.7 | 30.8 | 31.1 | 26.0 | 30.8 | 34.9 | 33.4 | 34.5 | 33.5 | 33.6 | 30.7 | 27.5 | 34.3 | | 34.0 | 32.1 | 31.9 | 34.9 | 34.2 |
|  | 29.7 | 31.6 | 28.0 | 49.0 | 33.9 | 30.8 | 32.4 | 29.5 | 29.9 | 32.2 | 36.7 | 47.6 | 33.0 | | 28.2 | 28.7 | 31.5 | 30.8 | 28.0 |
| Education (% of subgroup total) | 3.5 | 5.1 | 4.8 | 16.9 | 7.5 | 17.0 | 5.8 | 5.1 | 6.2 | 6.3 | 8.7 | 12.3 | 17.6 | | 16.2 | 15.6 | 15.4 | 17.0 | 15.6 |
|  | 36.1 | 39.5 | 40.6 | 45.1 | 44.6 | 43.8 | 37.3 | 38.2 | 39.4 | 40.7 | 43.4 | 45.5 | 41.7 | | 41.2 | 41.9 | 41.6 | 43.8 | 42.1 |
|  | 60.4 | 55.3 | 54.7 | 38.0 | 47.9 | 39.2 | 56.9 | 56.6 | 54.4 | 53.0 | 47.9 | 42.2 | 40.8 | | 42.7 | 42.5 | 43.1 | 39.2 | 42.3 |
| Civil Status (% of subgroup total) | 19.0 | 19.9 | 18.7 | 14.2 | 18.2 | 66.0 | 49.6 | 54.1 | 54.8 | 53.5 | 51.5 | 44.1 | 62.4 | | 68.2 | 70.2 | 67.3 | 66.0 | 69.7 |
|  | 81.0 | 80.1 | 81.3 | 85.8 | 81.8 | 34.0 | 50.4 | 45.9 | 45.2 | 46.5 | 48.5 | 55.9 | 37.6 | | 31.8 | 29.8 | 32.7 | 34.0 | 30.3 |
| Municipality group (% of subgroup total) | 24.7 | 23.9 | 24.4 | 23.2 | 24.7 | 32.2 | 29.5 | 29.1 | 29.8 | 28.0 | 28.2 | 25.1 | 33.8 | | 33.4 | 30.6 | 27.2 | 32.2 | 31.0 |
|  | 24.1 | 24.6 | 26.3 | 23.1 | 26.7 | 32.5 | 29.5 | 31.4 | 30.5 | 30.8 | 29.2 | 25.3 | 31.1 | | 32.8 | 32.2 | 28.8 | 32.5 | 32.0 |
|  | 51.1 | 51.6 | 49.2 | 53.7 | 48.6 | 35.2 | 41.0 | 39.5 | 39.7 | 41.2 | 42.5 | 49.5 | 35.1 | | 33.8 | 37.2 | 43.9 | 35.2 | 37.0 |

**Additional Table 3**. Trajectory group characteristics at baseline for females. Numbers indicate percent (%) of subgroup total if not otherwise specified.

| Age group (years) | | 1973-1987 | | | | | | 1953-1972 | | | | | | | 1938-1952 | | | | | |
| --- | --- | --- | --- | --- | --- | --- | --- | --- | --- | --- | --- | --- | --- | --- | --- | --- | --- | --- | --- | --- |
| Trajectory group | | **Group 1** | **Group 2** | **Group 3** | **Group 4** | **Group 5** | **Group 6** | **Group 1** | **Group 2** | **Group 3** | **Group 4** | **Group 5** | **Group 6** | **Group 7** | **Group 1** | **Group 2** | **Group 3** | **Group 4** | **Group 5** | **Group 6** |
| Age (mean) | | 27.6 | 27.6 | 27.8 | 26.9 | 26.8 | 27.7 | 43.6 | 44.6 | 45.1 | 44.2 | 43.6 | 45.1 | 45.5 | 60.7 | 61.5 | 62.0 | 61.1 | 61.8 | 62.3 |
| Income (% of subgroup total) | low income | 25.8 | 26.2 | 29.0 | 21.6 | 25.1 | 19.7 | 29.6 | 33.4 | 30.6 | 32.2 | 31.6 | 28.2 | 25.6 | 27.8 | 29.9 | 32.7 | 31.9 | 30.4 | 29.1 |
|  | medium income | 36.4 | 34.5 | 35.9 | 32.4 | 35.0 | 30.4 | 34.7 | 31.9 | 29.7 | 34.1 | 33.7 | 28.5 | 28.3 | 33.0 | 33.4 | 32.5 | 33.3 | 31.2 | 31.2 |
|  | high income | 37.8 | 39.2 | 35.1 | 46.1 | 39.9 | 50.0 | 35.7 | 34.8 | 39.6 | 33.8 | 34.6 | 43.3 | 46.0 | 39.1 | 36.7 | 34.7 | 34.8 | 38.4 | 39.7 |
| Education (% of subgroup total) | primary school | 2.7 | 5.2 | 3.3 | 6.4 | 3.2 | 9.4 | 3.9 | 4.1 | 5.1 | 3.6 | 3.7 | 5.9 | 7.0 | 19.4 | 18.7 | 16.5 | 16.0 | 16.0 | 15.3 |
|  | secondary school | 33.4 | 38.6 | 34.9 | 40.6 | 33.9 | 44.2 | 38.4 | 38.5 | 41.9 | 38.5 | 37.2 | 41.6 | 43.1 | 43.6 | 43.2 | 42.8 | 43.5 | 42.7 | 43.4 |
|  | higher education | 63.9 | 56.3 | 61.8 | 53.0 | 62.8 | 46.5 | 57.7 | 57.4 | 53.0 | 57.9 | 59.1 | 52.5 | 49.9 | 37.0 | 38.2 | 40.7 | 40.5 | 41.3 | 41.3 |
| Civil Status (% of subgroup total) | single | 28.8 | 29.3 | 31.8 | 22.6 | 23.9 | 26.7 | 56.1 | 56.3 | 53.5 | 58.1 | 56.1 | 50.8 | 48.3 | 61.2 | 62.9 | 63.2 | 62.6 | 60.6 | 58.9 |
|  | married/cohabitant | 71.2 | 70.7 | 68.2 | 77.4 | 76.1 | 73.3 | 43.9 | 43.7 | 46.5 | 41.9 | 43.9 | 49.2 | 51.7 | 38.8 | 37.1 | 36.8 | 37.4 | 39.4 | 41.1 |
| Municipality group (% of subgroup total) | urban | 25.8 | 22.5 | 24.3 | 22.1 | 23.3 | 23.5 | 32.5 | 27.4 | 26.8 | 30.1 | 28.4 | 24.5 | 21.4 | 33.6 | 33.3 | 30.5 | 30.5 | 26.6 | 23.2 |
|  | semi-urban | 26.4 | 25.0 | 25.8 | 23.7 | 23.3 | 23.7 | 32.3 | 29.8 | 28.9 | 31.3 | 31.7 | 27.5 | 24.8 | 32.0 | 32.5 | 31.9 | 31.8 | 29.8 | 27.7 |
|  | rural | 47.8 | 52.5 | 49.8 | 54.2 | 53.4 | 52.8 | 35.2 | 42.9 | 44.3 | 38.6 | 40.0 | 48.1 | 53.7 | 34.4 | 34.2 | 37.6 | 37.6 | 43.7 | 49.1 |

**Additional Table 4**. Bivariate logistic regression on male trajectory groups showing odds ratios (ORs) of change in SHC utilization due to differences in predisposing and enabling factors.

| Age group | | 20-34 | | 35-54 | | 55-69 | |
| --- | --- | --- | --- | --- | --- | --- | --- |
| Compared trajectory groups* | | Group 2 vs. 1 (ref.) | Group 3 vs. 5 (ref.) | Group 3 vs. 2 (ref.) | Group 5 vs. 4 (ref.) | Group 2 vs. 1 (ref.) | Group 3 vs. 5 (ref.) |
| Corresponding generic trajectory groups** | | I vs. V (ref.) | III vs. VI (ref.) | I vs. V (ref.) | II vs. III (ref) | I vs. V (ref.) | II vs. III (ref) |
| Analyzed utilization change | | OR for increase from low utilization | OR for decrease from intermediate utilization | OR for increase from low utilization | OR for increase vs. decrease from intermediate utilization | OR for increase from low utilization | OR for increase vs. decrease from intermediate utilization |
| Age | | 1 .00 (1.00-1.01) | 0.99 (0.98-0.99) | 1.06 (1.06-1.07) | 1.04 (1.03-1.04) | 1.07 (1.06-1.07) | 1.09 (1.08-1.10) |
| Income | low income | 1 | 1 | 1 | 1 | 1 | 1 |
|  | middle income | 0.98 (0.93-1.02) | 1.22 (1.15-1.29) | 1.09 (1.04-1.15) | 0.80 (0.77-0.84) | 1.16 (1.10-1.22) | 0.99 (0.92-1.07) |
|  | high income | 0.87 (0.83-0.91) | 1.41 (1.33-1.49) | 1.17 (1.11-1.23) | 0.84 (0.80-0.88) | 1.35 (1.28-1.42) | 1.23 (1.14-1.32) |
| Education | primary school | 1 | 1 | 1 | 1 | 1 | 1 |
|  | secondary school | 0.74 (0.68-0.82) | 1.42 (1.29-1.57) | 0.98 (0.90-1.07) | 0.77 (0.71-0.83) | 1.07 (1.01-1.14) | 1.04 (0.95-1.13) |
|  | higher education | 0.62 (0.57-0.68) | 1.79 (1.63-1.97) | 0.89 (0.81-0.97) | 0.65 (0.60-0.70) | 1.14 (1.07-1.21) | 1.18 (1.08-1.29) |
| Civil status | single | 1 | 1 | 1 | 1 | 1 | 1 |
|  | married or cohabitant | 1.06 (1.01-1.11) | 1.03 (0.97-1.09) | 1.23 (1.18-1.28) | 0.92 (0.89-0.96) | 1.29 (1.24-1.35) | 1.21 (1.14-1.29) |
| Municipality group | urban | 1 | 1 | 1 | 1 | 1 | 1 |
|  | semi-urban | 1.01 (0.96-1.06) | 0.97 (0.92-1.03) | 1.07 (1.02-1.13) | 0.92 (0.88-0.96) | 1.09 (1.04-1.15) | 0.94 (0.87-1.01) |
|  | rural | 0.96 (0.91-1.00) | 0.98 (0.93-1.03) | 1.05 (1.00-1.10) | 0.97 (0.93-1.02) | 1.02 (0.98-1.08) | 0.9 (0.84-0.97) |
| * as shown in Additional Figure 1 | | | | | | | |
| ** as shown in Figure 1 | | | | | | | |

**Additional Table 5**. Bivariate logistic regression on female trajectory groups showing odds ratios (ORs) of change in SHC utilization due to differences in predisposing and enabling factors.

| Age group (years) | | 20-34 | | | 35-54 | | | | 55-69 | | |
| --- | --- | --- | --- | --- | --- | --- | --- | --- | --- | --- | --- |
| Compared trajectory groups* | | Group 5 vs. 1 (ref.) | Group 4 vs. 3 (ref.) | Group 4 vs. 2 (ref.) | Group 4 vs. 1 (ref.) | Group 3 vs. 5 (ref.) | Group 3 vs. 2 (ref.) | Group 6 vs. 7 (ref.) | Group 2 vs. 1 (ref.) | Group 3 vs. 4 (ref.) | Group 5 vs. 6 (ref.) |
| Corresponding generic trajectory groups** | | I vs. V (ref.) | II vs. III (ref) | II vs. III (ref) | I vs. V (ref.) | II vs. III (ref) | II vs. III (ref) | IV vs. VII (ref.) | I vs. V (ref.) | II vs. III (ref) | IV vs. VII (ref.) |
| Analyzed utilization change | | OR for increase from low utilization | OR for increase vs. decrease from intermediate utilization | OR for increase vs. decrease from intermediate utilization | OR for increase from low utilization | OR for increase vs. decrease from intermediate utilization | OR for increase vs. decrease from intermediate utilization | OR for decrease from high utilization | OR for increase from low utilization | OR for increase vs. decrease from intermediate utilization | OR for decrease from high utilization |
| Age | | 0,96 (0,96-0,97) | 0,95 (0,94-0,95) | 0,96 (0,95-0,97) | 1,02 (1,02-1,02) | 1,05 (1,04-1,05) | 1,01 (1,01-1,02) | 0,99 (0,98-0,99) | 1,05 (1,04-1,05) | 1,05 (1,05-1,06) | 0,97 (0,96-0,98) |
| Income | low income | 1 | 1 | 1 | 1 | 1 | 1 | 1 | 1 | 1 | 1 |
|  | middle income | 0,91 (0,87-0,95) | 0,69 (0,64-0,74) | 0,80 (0,74-0,86) | 1,04 (1,00-1,08) | 0,77 (0,73-0,81) | 0,82 (0,78-0,86) | 1,07 (0,99-1,16) | 1,08 (1,02-1,14) | 0,98 (0,93-1,02) | 1,03 (0,96-1,11) |
|  | high income | 0,92 (0,88-0,96) | 0,57 (0,52-0,62) | 0,70 (0,64-0,76) | 1,15 (1,10-1,20) | 0,85 (0,80-0,89) | 0,81 (0,77-0,85) | 1,17 (1,08-1,27) | 1,15 (1,08-1,21) | 1,03 (0,98-1,07) | 1,08 (1,00-1,16) |
| Education | primary school | 1 | 1 | 1 | 1 | 1 | 1 | 1 | 1 | 1 | 1 |
|  | secondary school | 0,84 (0,74-0,94) | 0,61 (0,53-0,70) | 0,85 (0,74-0,98) | 1,07 (0,98-1,17) | 0,80 (0,72-0,90) | 0,88 (0,80-0,98) | 1,14 (0,99-1,31) | 1,03 (0,97-1,09) | 0,96 (0,91-1,01) | 0,94 (0,86-1,03) |
|  | higher education | 0,81 (0,72-0,90) | 0,45 (0,39-0,52) | 0,76 (0,66-0,88) | 1,07 (0,98-1,17) | 0,64 (0,58-0,71) | 0,75 (0,68-0,83) | 1,24 (1,08-1,43) | 1,07 (1,01-1,14) | 0,98 (0,92-1,03) | 0,96 (0,88-1,05) |
| Civil status | single | 1 | 1 | 1 | 1 | 1 | 1 | 1 | 1 | 1 | 1 |
|  | married or cohabitant | 0,78 (0,75-0,81) | 0,63 (0,58-0,68) | 0,70 (0,65-0,76) | 1,08 (1,05-1,12) | 0,90 (0,86-0,94) | 0,89 (0,85-0,93) | 1,11 (1,03-1,18) | 1,08 (1,03-1,13) | 1,02 (0,99-1,06) | 1,07 (1,01-1,14) |
| Municipality group | urban | 1 | 1 | 1 | 1 | 1 | 1 | 1 | 1 | 1 | 1 |
|  | semi-urban | 0,79 (0,75-0,83) | 0,84 (0,78-0,91) | 0,92 (0,85-0,99) | 0,88 (0,85-0,92) | 0,82 (0,78-0,87) | 0,94 (0,89-0,99) | 1,24 (1,14-1,34) | 1,02 (0,97-1,08) | 1,01 (0,96-1,05) | 1,21 (1,12-1,30) |
|  | rural | 0,81 (0,77-0,85) | 0,84 (0,77-0,9) | 0,95 (0,88-1,03) | 0,84 (0,81-0,88) | 0,85 (0,80-0,90) | 0,95 (0,90-1,00) | 1,28 (1,17-1,39) | 1,00 (0,95-1,05) | 1,00 (0,96-1,05) | 1,29 (1,20-1,39) |
| * as shown in Additional Figure 1 | | | | | | | | | | | |
| ** as shown in Figure 1 | | | | | | | | | | | |
